# Circuit Motifs for Contrast-Adaptive Differentiation in Early Sensory Systems: The Role of Presynaptic Inhibition and Short-Term Plasticity

**DOI:** 10.1371/journal.pone.0118125

**Published:** 2015-02-27

**Authors:** Danke Zhang, Si Wu, Malte J. Rasch

**Affiliations:** 1 Department of Biomedical Engineering, Hangzhou Dianzi University, Hangzhou, China; 2 State Key Laboratory of Cognitive Neuroscience and Learning, IDG/McGovern Institute for Brain Research, Beijing Normal University, Beijing, China; Georgia State University, UNITED STATES

## Abstract

In natural signals, such as the luminance value across of a visual scene, abrupt changes in intensity value are often more relevant to an organism than intensity values at other positions and times. Thus to reduce redundancy, sensory systems are specialized to detect the times and amplitudes of informative abrupt changes in the input stream rather than coding the intensity values at all times. In theory, a system that responds transiently to fast changes is called a differentiator. In principle, several different neural circuit mechanisms exist that are capable of responding transiently to abrupt input changes. However, it is unclear which circuit would be best suited for early sensory systems, where the dynamic range of the natural input signals can be very wide. We here compare the properties of different simple neural circuit motifs for implementing signal differentiation. We found that a circuit motif based on presynaptic inhibition (PI) is unique in a sense that the vesicle resources in the presynaptic site can be stably maintained over a wide range of stimulus intensities, making PI a biophysically plausible mechanism to implement a differentiator with a very wide dynamical range. Moreover, by additionally considering short-term plasticity (STP), differentiation becomes contrast adaptive in the PI-circuit but not in other potential neural circuit motifs. Numerical simulations show that the behavior of the adaptive PI-circuit is consistent with experimental observations suggesting that adaptive presynaptic inhibition might be a good candidate neural mechanism to achieve differentiation in early sensory systems.

## Introduction

Typically, sensory input signals vary slowly for most of the time and abrupt changes in value occur relatively rarely [1, 2]. Thus, when a sensory system attempts to efficiently code the input signals, abrupt changes are most important as they carry most of the useful information. Indeed, it has been found that early visual and olfactory systems tend to amplify the neural response to fast changes while remaining relatively silent for slow changes in the mean input intensity [1, 3–5]. In signal theory, systems with such response characteristics are called differentiators, because responses are proportional to changes in the input stream rather than to the mean intensity. In contrast, systems responding proportional to the (running) mean input intensity are called (leaky) integrators.

A number of different neural mechanisms exist and have been proposed that have the ability to respond strongly to the informative changes in the sensory input, while staying relatively silent to slowly varying, redundant inputs, and thus act as differentiators. In principle, to amplify a step increase in input intensity over the baseline steady state level, an excitatory feedforward drive has to be coupled with a (delayed) negative component that retracts the transient rise to a lower plateau. Roughly two kinds of neural mechanisms can achieve such a differentiator response: 1) inhibitory activity (circuit-based) and 2) purely synaptic modifications (synapse-based, i.e., without relying on inhibitory neurons). In circuit-based differentiators, the negative components are generated by inhibitory neurons, either by feed-forward inhibition [6, 7] or feedback inhibition [8]. In synapse-based mechanisms, synaptic properties are directly modulated in an input history dependent way, e.g. by synaptic depression [9–11].

While thus a differentiator can have different designs in neural circuitry, not all possible mechanisms might fit to the requirements of early sensory systems. In particular, a suitable differentiator for early sensory systems has to cope with the high dynamic range of natural signals, often spanning multiple orders of magnitude [12], much higher than the very limited dynamical range of single neurons [13, 14]. Moreover, there is some experimental evidence that neurons in early sensory systems need to adapt their temporal filtering properties to input contrast changes, with a longer and monophasic integration window for low contrast inputs and a narrower biphasic integration window for high contrast inputs [5, 15, 16]. Since a systematic comparison of the diverse mechanisms to implement a differentiator is lacking, it is unclear which of the possible mechanisms should be favored for early sensor systems.

In this study, we compare the response characteristics of a number of neural differentiator circuit motifs. In particular, we examine the contrast and intensity dependence of feedforward inhibition, feedback inhibition, and synaptic depression mechanisms. We find that *presynaptically* mediated feedforward inhibition functions in the widest dynamical range of all tested differentiator circuits. Crucially, despite the wide dynamical range, vesicle resources are maintained regardless of the input intensity, a property that is in stark contrast to the other circuit mechanisms investigated. This advantageous property might be the reason why presynaptic inhibition, an axo-axonic form of inhibition, is specifically observed in early sensory systems such as the retina [17, 18], in the lateral geniculate nucleus (LGN) [19], and in the olfactory system in drosophila [20–22]. Finally, we show that a presynaptic inhibition circuit can easily gain contrast adaptivity by additionally assuming short-term synaptic plasticity of the excitatory-to-excitatory neuron synapses. We thus conclude that presynaptic inhibition might be an important neural mechanism in early sensory systems, where high dynamic ranges and contrast adaptivity are important requirements.

## Methods

### The PI circuit model

We here introduce the mathematical details of our PI-circuit model.

#### Network structure

**Fig 1:**
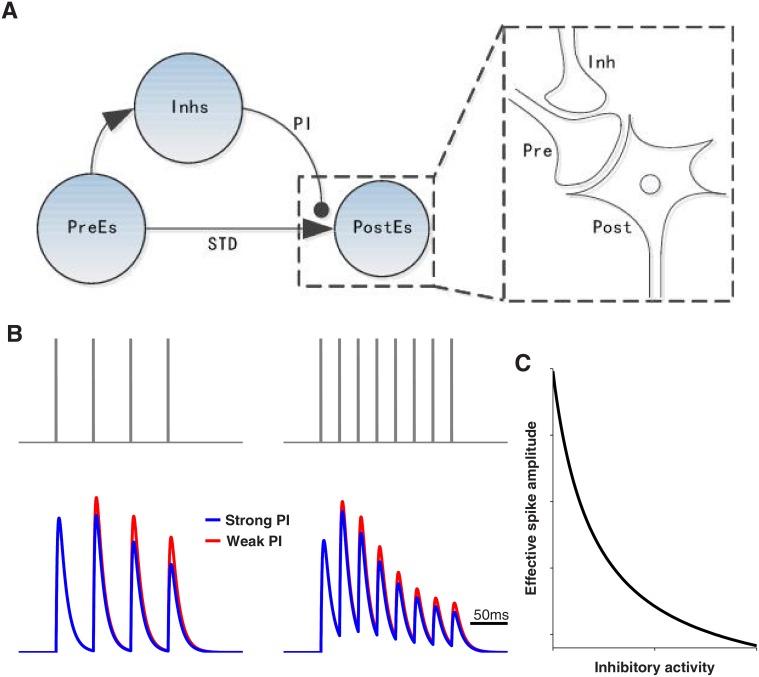
Pre-synaptic inhibition circuit with synaptic short-term plasticity. A: Circuit structure with an illustration of the axo-axonic connection mediating presynaptic inhibition (PI; see inset). B: Effect of short-term plasticity of the **PreE**-to-**PostE** synapses for two different input spike rates. Note that synaptic conductances show early facilitation and late depression. Pre-synaptic inhibition (PI) additionally modulates the synaptic efficacy (red versus blue line). C: The effective spike amplitude *p* within the **PreE**-to-**PostE** synapses is reduced by inhibitory activity.

We consider three populations of neurons, namely, presynaptic excitatory neurons (**PreE**), receiving the input signal, postsynaptic excitatory neurons (**PostE**), representing the output signal, and inhibitory neurons (**Inh**), see illustration in Fig. 1. Populations **PostE** and **Inh** both receive connections from the **PreE** neurons. In the PI-circuit, **PostE** neurons are presynaptically inhibited by **Inh** neurons, that is, the axon terminal of an **Inh** neuron targets the presynaptic site of a **PreE**-to-**PostE** synapse. For simplicity of the investigation of the dynamics of the circuit motifs, we assume that there are no recurrent interactions within the same type of neurons.

#### Single neuron dynamics

Each single neuron is modeled as a leaky-integrate-and-fire (LIF) neuron (see e.g. [23]). That is, when the membrane potential of a neuron is above a spiking threshold, an action potential is initiated. After the spike, the membrane potential is immediately reset to the resting membrane potential level. Below the spiking threshold, the dynamics of a neuron is given by

$$\begin{array}{c} C^{T}\frac{dv_{i}^{T}}{dt}=-g_{L}^{T}\left(v_{i}^{T}-E_{L}^{T}\right)-I_{i}^{T}, \end{array}$$ (1)

where *C* is the membrane capacitance, and T∈{E,I} denotes the neuron type, excitatory or inhibitory, respectively. *g*_*L*_ is the leak conductance, and $E_{L}$ the corresponding reversal potential. For simplicity, parameters are chosen identical for both neuron types (see Table 1 for an overview of all parameter values used in the simulations in the Results section).

**Table 1:** Parameter values used in the numerical simulations. It is T∈{E,I}.

**Description**	**Parameter**	**Values**
Number of **PreE** neurons	*N* ^ext^	400
Number of **PostE** neurons	$N^{\text{E}}$	50
Number of **Inh** neurons	$N^{\text{I}}$	100
Membrane capacitance	$C^{\text{T}}$	600pF
Leakage conductance	$g_{L}^{\text{T}}$	30nS
Resting potential	$E_{L}^{\text{T}}$	-70mV
Excitatory reversal potential	$E_{\text{E}}$	0mV
Firing threshold	*V*_*th*_	-50mV
Mean of excitatory background noise	$\mu^{\text{E}}$	7.5nS
Mean of inhibitory background noise	$\mu^{\text{I}}$	0nS
Variance of background noise	*σ*	1
Slope of I-O function	$\alpha^{\text{T}}$	4.79
Threshold of I-O function	$\beta^{\text{T}}$	4.63nS
**PreE**-**PostE** synaptic connection strength	$w^{\text{EE}}$	0.6nS
**PreE**-**Inh** synaptic connection strength	$w^{\text{IE}}$	0.4nS
**Inh**-**PostE** synaptic connection strength	$w^{\text{I}}$	0.1nS
Time constant for excitatory synaptic current	$\tau^{\text{E}}$	5ms
Time constant for inhibitory synaptic current	$\tau^{\text{I}}$	10ms
Time constant for synaptic depression	$\tau_{\text{D}}$	300ms
Time constant for synaptic facilitation	$\tau_{\text{F}}$	80ms
Time constant for presynaptic inhibition	$\tau_{\text{P}}$	300ms
Short-term depression strength	*U*	0.25
Time constant for **PostE** firing rate	*τ*_*r*_	10ms
Time constant for **PostE** firing rate	*τ*_*r*_	10ms
Time constant for excitatory current	*τ*_*exc*_	50ms
Time constant for inhibitory current	*τ*_*inh*_	100ms
Connection strength	w	0.3
Connection strength	A	400
Range of sensory input(low contrast)	[*n*_*min*_, *n*_*max*_]	[8.5, 9.5]
Range of sensory input(high contrast)	[*n*_*min*_, *n*_*max*_]	[5, 13]

Since the inhibition here acts on the presynaptic terminal of excitatory connections, all direct synaptic interactions in the PI-circuit are thus excitatory. Synaptic currents received by the *i*-th T-type neuron is thus given by

$$\begin{array}{c} I_{i}^{T}=g_{i}^{TE}\left(v_{i}^{T}-E_{E}\right)+\xi \left(v_{i}^{T}-E_{E}\right), \end{array}$$ (2)

where $g_{i}^{\text{TE}}$ is the excitatory conductance received by the *i*th T-type neuron, $E_{\text{E}}=0mV$ is the reversal potential of excitatory current, and *ξ* denotes a Gaussian noise process characterized by mean *μ* ^*T*^ and variance *σ* ^2^.

We assume for simplicity that all synaptic input connections to a particular neuron have identical strength. In this case, for an inhibitory neuron, the dynamics of the excitatory conductance $g_{i}^{\text{IE}}$ is given by

$$\begin{array}{c} \frac{dg_{i}^{IE}}{dt}=-\frac{g_{i}^{IE}}{\tau^{E}}+w^{IE}\sum_{j}\sum_{l}\delta \left(t-t_{jl}^{E}\right), \end{array}$$ (3)

where $w^{IE}$ is the connection strength from a **PreE** to an **Inh** neuron, and $\tau^{E}$ is the synaptic time constant. The synaptic conductance $g_{i}^{\text{IE}}$ is driven by the activity of the **PreE** neurons, whose spike trains are generated according to Poisson processes; $t_{jl}^{\text{E}}$ denotes the moment of the *l*th spike of the *j*th **PreE** neuron.

Similarly, the overall synaptic conductance received by a **PostE** neuron from the **PreE** neurons, $g_{i}^{\text{EE}}$, is given by the sum of the individual conductances

$$\begin{array}{c} g_{i}^{EE}=\sum_{j}g_{ij}^{EE}. \end{array}$$ (4)

In contrast to the **PreE**-to-**Inh** synapses, however, the dynamic of the **PreE**-to-**PostE** synapses now incorporates presynaptically mediated inhibition and short-term synaptic plasticity. Thus, the conductance change upon arrival of a presynaptic spike is determined by the spike amplitude at presynaptic terminal *p*(*t*) (which is affected by inhibition), the available vesicle resource *x*(*t*), the vesicle release probability *u*(*t*) and the overall synaptic connection strength $w^{EE}$. Hence,

$$\begin{array}{c} \frac{dg_{ij}^{EE}}{dt}=-\frac{g_{ij}^{EE}}{\tau^{E}}+w^{EE}u_{ij}\;x_{ij}\;p\sum_{l}\delta \left(t-t_{jl}^{E}\right). \end{array}$$ (5)

#### Presynaptic inhibition

The **PreE**-to-**PostE** synapses are presynaptically modulated by the activities of the **Inh** neurons. Presynaptic inhibition is known to shunt the membrane potential in the presynaptic terminal for each spike individually [24]. Thus, inhibitory activity modifies the “effective spike amplitude”, i.e. the effective height of the voltage change caused by a presynaptic excitatory spike arriving at the pre-synaptic terminal.

To model this synaptic modulation effect, we assume that the effective spike amplitude *p* is a monotonically decreasing function of the inhibitory activity (see Fig. 1C). Since dendro-dendritic interactions among inhibitory neurons in early sensory systems will cause inhibitory activity to be very correlated [25, 26], we further assume that presynaptic inhibition in one neuron is mediated by the total average inhibitory activity received. Thus, we have $s=\frac{1}{N^{\text{I}}}\sum_{k}s_{k}$, with the synaptic dynamics *s*_*k*_ of an inhibitory synapse *k*

$$\begin{array}{c} \frac{ds_{k}}{dt}=-\frac{s_{k}}{\tau^{I}}+w^{I}\sum_{l}\delta \left(t-t_{kl}^{I}\right), \end{array}$$ (6)

where $\tau^{I}$ is a time constant and $t_{kl}^{\text{I}}$ denotes the time of the *l*th spike of inhibitory neuron *k*.

Taken together, the dynamics of the effective spike amplitude is

$$\begin{array}{c} \tau_{p}\frac{dp}{dt}=-p+\frac{1}{1+\rho \;\frac{1}{N^{I}}\sum_{k}s_{k}}, \end{array}$$ (7)

where *ρ* and *τ*_p_ are parameters characterizing the dynamics of presynaptic inhibition.

#### Synaptic short-term plasticity

Since a fraction of the available synaptic vesicles will be consumed for each presynaptic spike, the number of the currently available vesicles in the synapse terminal depends on the recent spike history. Because the recovery of the vesicle resource is a relatively slow process, the usable fraction of the vesicle resources for each spike arrival decreases, resulting in short-term synaptic depression. On the other hand, since spikes induce calcium influx and accumulated calcium up-regulates the vesicle release probability, synaptic efficacy can facilitate as well. Synaptic facilitation and depression often co-exist in the same synapse showing an early phase of facilitation and a later phase of depression (see Fig. 1B). We follow [27] to model this short-term synaptic plasticity and thus have

$$\begin{array}{c} \frac{dx_{ij}}{dt}=\frac{1-x_{ij}}{\tau_{D}}-x_{ij}\;u_{ij}\;p\sum_{l}\delta \left(t-t_{jl}^{E}\right), \end{array}$$ (8)

$$\begin{array}{c} \frac{du_{ij}}{dt}=-\frac{u_{ij}}{\tau_{F}}+U\left(1-u_{ij}\right)\;p\sum_{l}\delta \left(t-t_{jl}^{E}\right), \end{array}$$ (9)

where *x*_*ij*_ denotes the available vesicle resource in the presynaptic terminal of a *j*th **PreE** neuron (making a synapse onto the *i*th **PostE** neuron), and *u*_*ij*_ the corresponding vesicle release probability. In contrast to previous models, however, the STP variables here are dependent on the presynaptically controlled “effective spike amplitude” *p*. The constants $\tau_{D}$ and $\tau_{F}$ are the time scales of the recovery of the vesicle resource and the release probability, respectively. For a synapse with strong short-term depression, recovery of vesicle resource is slower than facilitation [28], i.e. $\tau_{D}>\tau_{F}$. In summary, the fraction of vesicle resource consumed for each spike depends on the product of available vesicle resources, the release probability *u* and the effective spike amplitude *p*.

#### Mean-field analysis

We use the well-known mean-field analysis to approximate the slow dynamics of the neural circuit [29, 30]. We assume that the input-output function of each neuronal population is of linear-threshold type

$$\begin{array}{c} r^{T}=\alpha^{T}{\left[g^{TE}-\beta^{T}\right]}_{+}, \end{array}$$ (10)

where $r^{\text{T}}$, T∈{E,I}, is the average neuronal population firing rate of **PostE** or **Inh** neurons, respectively. The slope $\alpha^{\text{T}}$ and the threshold $\beta^{\text{T}}$ characterize the input-output function of the neural population. In the mean field approximation, the average excitatory synaptic conductance received by the **Inh** and **PostE** populations are

$$\begin{array}{c} g^{IE}={\tilde{w}}^{IE}r \end{array}$$ (11)

$$\begin{array}{c} g^{EE}={\tilde{w}}^{EE}\;u\;x\;p\;r, \end{array}$$ (12)

where ${\tilde{w}}^{\text{TE}}\equiv w^{\text{TE}}\tau^{\text{E}}N^{\text{ext}}$ is a shortcut for the effective weight strength, and *r* is the firing rate of the input neural population **PreE**. The dynamics for the slow variables are

$$\begin{array}{c} \frac{dx}{dt}=\frac{1-x}{\tau_{D}}-x\;u\;p\;r \end{array}$$ (13)

$$\begin{array}{c} \frac{du}{dt}=-\frac{u}{\tau_{F}}+U\left(1-u\right)\;p\;r \end{array}$$ (14)

$$\begin{array}{c} \tau_{p}\frac{dp}{dt}=-p+\frac{1}{1+\rho s}, \end{array}$$ (15)

where the average inhibitory drive onto the presynaptic terminals is given by

$$\begin{array}{c} s={\tilde{w}}^{I}r^{I}, \end{array}$$ (16)

and $r^{\text{I}}$ is the rate of the **Inh** population and ${\tilde{w}}^{\text{I}}\equiv w^{\text{I}}\tau^{\text{I}}$ the effective connection strength.

From the Eqs. 13–15, we compute the firing rate response with respect to the **PreE** neurons’ firing rates. In the stationary state, it is

$$\begin{array}{c} \bar{x}=\frac{1}{1+\tau_{D}\bar{u}\;\bar{p}\;r} \end{array}$$ (17)

$$\begin{array}{c} \bar{u}=\frac{U\tau_{F}\;\bar{p}\;r}{1+U\tau_{F}\bar{p}\;r} \end{array}$$ (18)

$$\begin{array}{c} \bar{p}=\frac{1}{1+\rho \;{\tilde{w}}^{I}r^{I}}. \end{array}$$ (19)

Combining with Eqs.10–12 and Eq. 16, we find

$$\begin{array}{c} g^{EE}=\frac{{\tilde{w}}^{EE}\bar{p}r}{1+\tau_{D}\bar{p}r+1/\left(U\tau_{F}\;\bar{p}r\right)} \end{array}$$ (20)

$$\begin{array}{c} \bar{p}=\frac{1}{1+\rho \;{\tilde{w}}^{I}\alpha^{I}{\left[{\tilde{w}}^{IE}r-\beta^{I}\right]}_{+}} \end{array}$$ (21)

$$\begin{array}{c} r^{E}=\alpha^{E}{\left[g^{EE}-\beta^{E}\right]}_{+}. \end{array}$$ (22)

Thus, given input rate *r*, we can use Eqs. 20–22 to compute the steady state output rate $r^{\text{E}}$ of the circuit.

Note that we are here interested only in the stationary state response of the system, as well as the transient dynamics to reach the steady state. Thus we implicitly assume that the system parameters are set in a manner that no self-sustained oscillatory activity occurs, which would be biologically unrealistic for an early sensory system. Robustness against oscillation could be further analyzed with methods from system theory (e.g. [31]), but is beyond the scope of this study.

### Circuit dynamics for alternative circuit motifs

To compare the PI-circuit with alternative circuit motifs, we simulated a feedforward circuit, a feedback inhibitory circuit, and a postsynaptic feedforward inhibitory circuit with or without short-term plasticity.

In case of the purely excitatory feedforward circuit, we used the mean-field approximation as described above (with or without short-term depression), but clamped the inhibitory currents to zero.

For the feedback inhibitory circuit and postsynaptic feedforward inhibitory circuit, the circuit dynamics is given by

$$\begin{array}{ccc} \tau_{r}\frac{dr}{dt} & = & -r+I_{\text{exc}}-I_{\text{inh}} \end{array}$$ (23)

$$\begin{array}{c} \tau_{\text{exc}}\frac{dI_{\text{exc}}}{dt}=-I_{\text{exc}}+AuxI \end{array}$$ (24)

$$\begin{array}{c} \tau_{\text{inh}}\frac{dI_{\text{inh}}}{dt}=-I_{\text{inh}}+J \end{array}$$ (25)

$$\begin{array}{c} \frac{dx}{dt}=\frac{1-x}{\tau_{D}}-uxI \end{array}$$ (26)

$$\begin{array}{c} \frac{du}{dt}=-\frac{u}{\tau_{F}}+U\left(1-u\right)I. \end{array}$$ (27)

where *τ*_*r*_, *τ*_exc_ and *τ*_inh_ are time constants of the postsynaptic neuron’s firing rate, the excitatory current *I*_exc_ and the inhibitory current *I*_inh_, respectively. *I* represents the current input from presynaptic neurons, and *J* denotes the current received by the inhibitory neurons. For a feedforward synapse without short-term plasticity, we set *Aux* = 1 to be constant, where *A* is a parameter related to the strength of synaptic connection. For the feedback inhibitory circuit, *J* is driven by postsynaptic excitatory neurons, i.e., *J* = *wr*, with connection strength *w*. For the postsynaptic feedforward inhibitory circuit, inhibition *J* is driven by the sensory input, i.e., *J* = *I*.

Note that for the feedback inhibitory circuit and postsynaptic feedforward inhibitory circuit, the inhibitory synapses act directly on the postsynaptic neuron, which is different from the PI-circuit. For case of postsynaptic inhibition, the dendritic integration rule of simultaneously arriving excitatory and inhibitory currents could be very complicated and critically depends on the spatial location of inhibitory and excitatory inputs on the dendrites of the postsynaptic neuron [32]. We here simply assume that excitation and inhibition are spatially scattered at different dendritic branches, so that in first approximation excitatory and inhibitory currents are linearly integrated (see Eq. 23).

### Temporal filter estimation

For step inputs to the circuit, i.e. an abrupt increase from an input *r* = *r*_1_ to *r* = *r*_2_ at time *t* = 0, we use a simple direct method to estimate the causal filter *D* of the neural circuit in case of discrete time bins. Since a step input is constant apart from the abrupt change, the *i*th time bin of neural response after abrupt input change can be represented as

$$\begin{array}{c} r^{E}\left(i\right)=\frac{\left(r_{2}-r_{1}\right)}{r_{1}}\sum_{j=0}^{j=i}D(j)+r_{0}^{E}, \end{array}$$ (28)

where $r_{0}^{\text{E}}$ denotes the baseline firing rate of the **PostE** neurons, and *D*(*j*) represents the *j*-th component of the filter. Rewriting the above equation in matrix form, we find

$$\begin{array}{c} D=\frac{r_{1}}{r_{2}-r_{1}}R^{-1}\left(r^{E}-r_{0}^{E}\right), \end{array}$$ (29)

where *R* is a lower triangular matrix with each non-zero element equaling to 1.

### Linear-nonlinear characterization

To compare model responses with experimental results on contrast adaptation, we adopt similar stimuli as in the experiments [15]. We then fit the response with a linear-nonlinear (LN) method to characterize the circuit behavior as done in [15]. Briefly, the LN method predicts neural responses by assuming that a neuron can be approximated by a linear filter (the receptive field) convolving the input stream and a subsequent non-linear function (the activation function). Although all intrinsic biophysical dynamics are neglected, this simple model has the advantage that its parameters can be easily fitted. Thus, the LN method has been widely used to characterize the response properties of single neuron in experimental literature.

To fit such a LN model to the response of our circuit motifs, we followed [15] and first constructed a modulation signal *n*(*t*) for each time step by drawing from an uniform distribution in the range [*n*_min_, *n*_max_]. To ensure a large dynamic input range this modulation signal was then given to our model in exponentiated form according to

$$\begin{array}{c} r\left(t\right)=2^{n(t)}. \end{array}$$ (30)

Then, the filter *F* and nonlinearity *N* of the LN model, *N*(*F* * *n*(*t*)), was then fitted to the circuit motif response $r^{\text{E}}$ for different contrasts. Contrast levels were set by changing the range [*n*_min_, *n*_max_] of the modulation signal (see Table 1 for values used). Note that this definition of contrast is slightly different from above because here contrast refers to the variance of the input signal.

The estimation of the filter *F* was done as follows (see e.g. [15]). The modulation signal *n*(*t*) and the circuit response $r^{\text{E}}$ were first adjusted to have zero mean, and the linear filter was then estimated by computing the correlation between *n*(*t*) and response $r^{\text{E}}$, normalized by the autocorrelation of *n*(*t*), which can be done in the Fourier domain:

$$\begin{array}{c} F\left(\omega \right)=\frac{\left\langle {\tilde{n}}^{*}\left(\omega \right){\tilde{r}}^{E}\left(\omega \right)\right\rangle }{\left\langle {\tilde{n}}^{*}\left(\omega \right)\tilde{n}\left(\omega \right)\right\rangle } \end{array}$$ (31)

Here $\tilde{n}(\omega )$ is the Fourier transform of *n*(*t*), and ${\tilde{n}}^{*}(\omega )$ is the complex conjugate, and ⟨⋅⟩ denotes averaging over *t*. The estimated filter *F* was then normalized that the variance of the LN model response was equal to the variance of the input [15], i.e.,

$$\begin{array}{c} \text{Var}(n)=\text{Var}(h), \end{array}$$ (32)

where *h* = *F* * *n*(*t*) is the linearly filtered response. The nonlinearity *N*(*h*) was then calculated by examining the relation of $r^{\text{E}}$ and *h*.

## Results

### Comparison of mechanisms for difference detection

**Fig 2:**
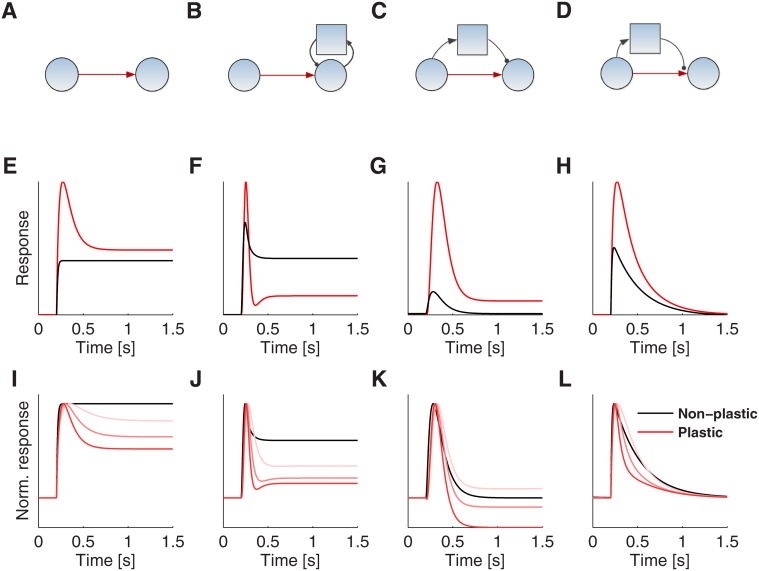
Comparison of different circuits in response to a step current input. A–D: Four circuits are compared: (A) a feedforward circuit, (B) a postsynaptic circuit with feedback inhibition, (C) a postsynaptic feedforward inhibition circuit, and (D) a presynaptic feedforward inhibition circuit. E–H: Exemplary responses of the circuits above to a step input, if the **PreE**-to-**PostE** synapse (red arrow in A–D) is subject to short-term plasticity (red lines) or not (black lines). I–L: Normalized circuit responses to steps of different contrast. Note that the normalized responses to different input contrasts are identical when the **PreE**-to-**PostE** synapse is not plastic (black lines). However, if plastic, transient response dynamics are contrast dependent (lines in shades of red). Note that only circuit D always returns to baseline levels even in case of different input contrasts.

A number of circuit motifs are capable of specifically amplifying sudden increases in the input signal intensity. We start with a comparison of the basic response properties of 4 generic circuit motifs that show transient responses to abrupt input changes and thus act (at least in part) as a differentiator system (illustrated in Fig. 2). In all circuit motifs a presynaptic excitatory neural population, **PreE**, receives external sensory inputs, feedforwardly connects to a post-synaptic excitatory population, **PostE** (excitatory populations are symbolized by circles in Fig. 2 A–D). If no negative feedback is available, this simple feed-forward motif will not response transiently to changes (see black line in Fig. 2E). There are in principle two ways to induce a transient response to changes, i.e. history-dependent synaptic modifications (Fig. 2A) or including inhibitory neurons (Fig. 2 B–D). If the excitatory feed-forward synapse **PreE**-to-**PostE** in Fig. 2A exhibits short-term plasticity (STP), an abrupt input change can cause a transient increase in the response level. Thus STP can be the basis of a differentiator system that amplifies a sudden increase in intensity over changes in the mean intensity level (Fig. 2E; red-line).

Alternatively, inhibitory neurons can cause transient response amplifications to sudden input changes in respect to the steady state response. In the simplest cases, inhibition can be driven feed-forwardly (Fig. 2C), or via feedback (Fig. 2B). The response of both circuits show transient amplification to input changes (black lines in Fig. 2F and G). Note, that parameters in the feedforward circuit can be adjusted, so that the response level after an input step returns to the identical steady state value as before the input change, although the input intensity is actually different. We call a system showing this response behavior a *perfect differentiator*. In contrast, in case of a feedback inhibitory circuit (Fig. 2B), inhibition grows only proportional to the excitatory response (and not proportional to the excitatory input currents), so that a complete balance of excitatory and inhibitory currents cannot be realized; perfect differentiation is not possible with feedback inhibition.

If the synapse **PreE**-to-**PostE** is made plastic (indicated by red-color in Fig. 2A–D; see Methods), the transient dynamics of the circuits changes with input contrast, a property suggested for early sensory systems [15]. Circuit responses to three different input contrast changes are shown in Fig. 2I–L. The amplitude of the transient response is proportional to the contrast in all circuits; the steady state attained after the transient response, however, changes as well (Fig. 2I–K). In particular, the perfect differentiation property of the feed-forward circuit is lost (Fig. 2K).

To overcome this drawback, we here propose an alternative circuit motif to achieve perfect differentiation across all input contrasts (Fig. 2D). As in the feed-forward inhibition circuit (Fig. 2C), an inhibitory population, **Inh**, receives input from the presynaptic neuron pool. However, the axon terminal of the **Inh** population acts on the presynaptic site of the synapse (instead of acting in the “normal”, postsynaptic manner) and thus divisively modulates its strength (see illustration in Fig. 1). This type of inhibition is called presynaptic inhibition (PI). We found that PI is an effective mechanism to implement a perfect differentiator in neural circuits (Fig. 2H, black line), even if adaptivity is incorporated with STP (Fig. 2H, red line). Moreover, the transient response is contrast adaptive while maintaining the perfect differentiator property (Fig. 2L; see mathematical analysis below).

There is good evidence that neurons in the sensory system can have transient responses to stimulus changes while ignoring the absolute value of input intensities [5, 33–35]. As shown below, a perfect differentiator is able to function in a wide range of input intensity because the steady state responses never saturate.

### Perfect difference detection in the PI-circuit

In the following, we investigate in mathematical detail the neural information processing capabilities of the PI circuit. In particular, we are interested in its temporal filtering properties and its contrast adaptability.

**Fig 3:**
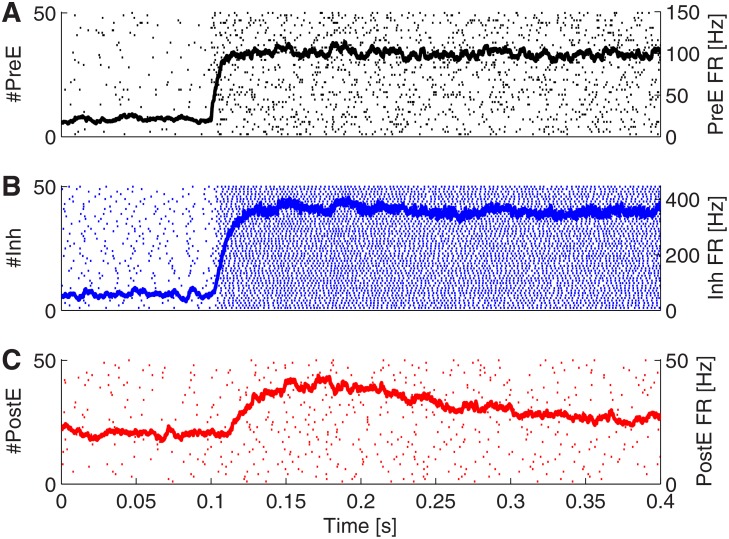
Neuronal spiking activities to step input. Firing rates of the **PreE** (A), **Inh** (B) and **PostE** (C) neuron populations in response to a step current input. Each dot in the background indicates a spike. Note that **PostE** shows a transient response to a step current input.

Assume that the (Poissonian) input firing rate *r* increases abruptly at time *t*_0_ from *r*_1_ to *r*_2_. In Fig. 3, the response of the PI-circuit to such an abrupt increase in the input firing rate is illustrated. When modeled in a network of Leaky-integrate-and-fire neurons (see Methods), the **PostE** neurons respond with several spikes closely after the onset of stimulus. Then, after this transient response, activity slowly relaxes to a stationary state. Using mean-field analysis [29, 30], we estimated the steady state of the **PostE** population firing rate $r^{\text{E}}$ as a function of the input rate *r*, which is given by Eqs.20–22.

**Fig 4:**
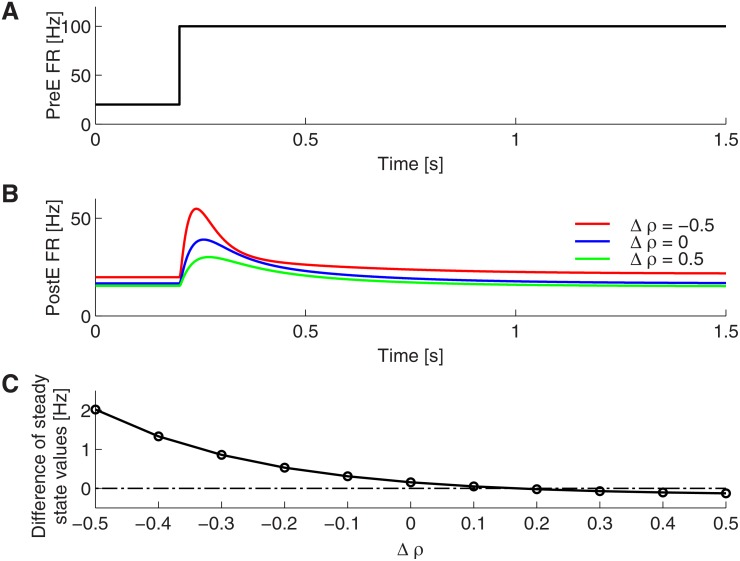
Differentiation by presynaptic inhibition. A–B: In response to a step input (A), **PostE** neurons show a transient response for different strengths of presynaptic inhibition and arrive at a new steady state level (B). C: Perfect differentiation, that is, the steady state level is the same as the initial baseline level, can be achieved when presynaptic inhibition strength is properly adjusted, i.e., here V* = 0.9.

Using this mean-field approximation, we can further analytically compute the transient firing rate response, as shown in Fig. 4A–B. We found that the output firing rate is a sigmoidal function of the product $\bar{p}r$, that is the input firing rate times the effect of PI on the spike amplitude $\bar{p}$. Varying the strength of the PI by changing the parameter *ρ*, the **PostE** neurons display different kinds of transient activities and steady state values (Fig. 4C). For weak PI, **PostE** decays to a state where its firing rate is slightly larger than the baseline level. When PI is very strong, the stationary state firing rate can even reach values below the initial baseline level.

Interestingly, in the special case when the parameter governing the strength of PI, *ρ*, is set to

$$\begin{array}{c} \rho^{*}=\frac{1}{{\tilde{w}}^{I}\alpha^{I}\beta^{I}}, \end{array}$$ (33)

the **PostE** neurons’ firing rate becomes insensitive to the absolute value of input firing rate. In other words, excitation and inhibition are balanced in such a way that after a transient response, the firing rate of the **PostE** neurons will return to the same baseline level as before the rate change. As explained above, we call this behavior of the circuit a perfect differentiation. Mathematically, we find that in the case of *ρ* = ρ*, it follows that $\bar{p}r=\beta^{I}/{\tilde{w}}^{I}E$ is a constant and independent from the input rate as soon as the inhibitory activity is driven above threshold, ${\tilde{w}}^{IE}r>\beta^{I}>0$. Note that in this case, the output firing rate is also independent from the input rate, because the steady state conductance $g^{\mathrm{EE}}$ is only dependent on the constant product $\bar{p}r$ (see Eq. 20). Interestingly, the vesicle resource and release probability becomes insensitive to **PreE** firing activity in case of *ρ* = ρ* (see Eqs. 17–18)), because of the transmitter release is presynaptically modulated by inhibitory activity. Therefore, this form of inhibition can maintain vesicle resources regardless of the presynaptic firing rate.

### The transient response obeys the Weber-Fechner law

**Fig 5:**
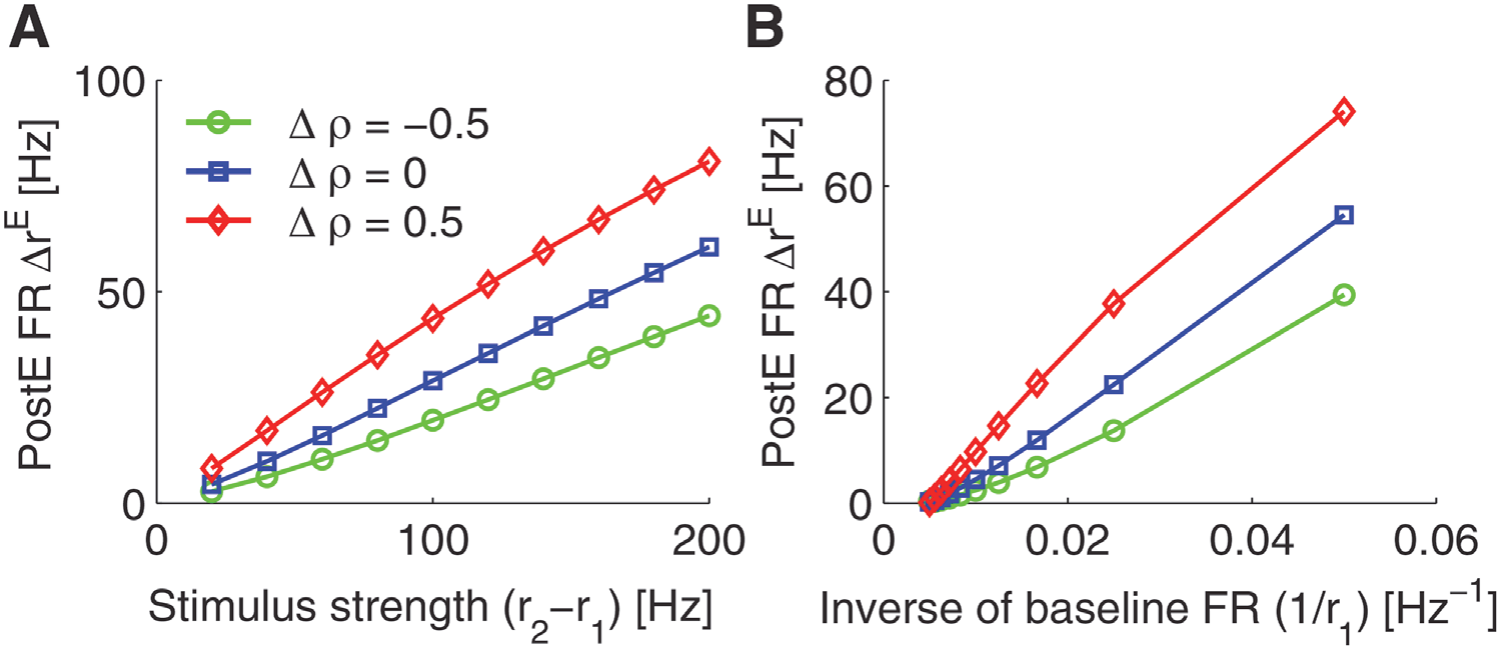
The transient response of the PI circuit obeys the Weber-Fechner Law. A: Stimulus strength is varied and the output rate of the PI circuit is plotted for different strengths of presynaptic inhibition. Note that only in the case of Δ*ρ* = 0, the transient response amplitude exhibits a linear relationship with input rate change when the baseline input rate is fixed. B: When the input rate change is fixed, the transient response amplitude is largely proportional to the inverse of the baseline firing rate. As in A, the linear relation only holds exactly true, if the presynaptic strength is adjusted to Δ*ρ* = 0.

When the external stimulus abruptly increases from *r*_1_ to *r*_2_, the firing rate of **PostE** neurons exhibits a transient dynamics. In case of the perfect differentiator setting, the response amplitude of the transient dynamics $\Delta r^{\text{E}}$ is proportional to the relative increase of the input firing rate, i.e., $\Delta r^{\text{E}}\propto \Delta r/r_{1}\equiv (r_{2}-r_{1})/r_{1}$. In Fig. 5A, we plot the $\Delta r^{\text{E}}$ versus the relative input change while fixing the baseline *r*_1_ and find a linear relationship. If instead Δ*r* is fixed and the baseline *r*_1_ is changed, we also find a linear relationship when $\Delta r^{\text{E}}$ is plotted as a function of 1/*r*_1_ (see Fig. 5B). The fact that the neural response amplitude is proportional to the relative amplitude of the sensory inputs is known as the Weber-Fechner law and a very common property among sensory system [36, 37].

We thus have shown that the behavior of the circuit model obeys the Weber-Fechner law. This result can be explained mathematically by examining the dynamical equations of our model. According to Eq. 10 and Eq. 12, the **PostE** neurons’ transient response shortly after the stimulus onset depends only on the input rate change, i.e. $\Delta r^{\text{E}}\propto \Delta g^{\text{EE}}={\tilde{w}}^{\text{EE}}uxp\Delta r\propto \Delta r$, since the STP related variables *u* and *x* as well as the PI variable *p* all change slowly and are thus approximately constant on a fast time scale. Moreover, shortly before the stimulus onset, the effective spike amplitude *p* is inversely proportional to the baseline firing rate, i.e., *p* ∝ 1/*r*_1_. Taken together, we thus have $\Delta r^{\text{E}}\propto (r_{2}-r_{1})/r_{1}$. This is just the requirement of the Weber-Fechner law.

By following the Weber-Fechner law, the PI-circuit is thus able to compress the wide dynamic range of inputs into the limited dynamic range of single neurons. How is this property useful for processing natural inputs? From a statistical point of view, natural signals cover a large dynamical range and thus can be described with distributions having long tails. Specifically, it has been suggested that a Student-t distribution can well model the statistics of naturally occurring signals [38, 39]. On the other hand, single neurons might not be able to cope with long-tailed distributions directly. For instance, popular linear-nonlinear Poisson models instead commonly assume input signals of a Gaussian form and find good fits to neural responses in the cortex [40, 41]. It is thus reasonable to assume that input signals having a long-tailed statistics have to be transformed to a statistical distribution that is more intelligible to neural systems, i.e. to a Gaussian-like form. Thus the question remains, how then the long-tailed natural signals become “gaussianized” by the early sensory systems to meet the limited capabilities of upstream cortical neurons?

**Fig 6:**
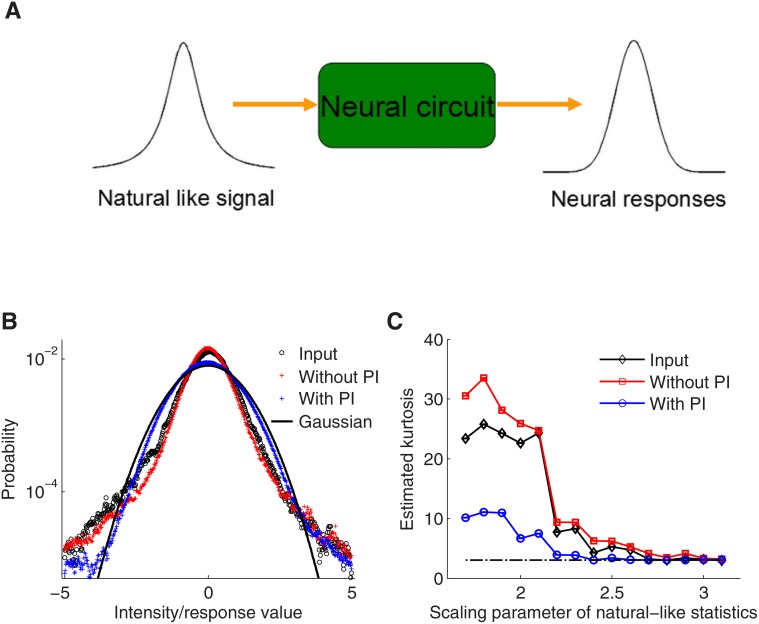
Gaussianization of sensory input signals by presynaptic inhibition. A: Schematic diagram of the setup. Natural signal like input distributions are converted by the neural circuit. The circuit causes that the resulting output distributions (i.e. synaptic conductance distributions to be processed further by upstream neural circuits) become differently shaped from the input distribution. We here analyze how far the output distribution deviates from a Gaussian shape in the cases of circuits with divisive or subtractive inhibition. B: Normalized distributions (zero mean and unit variance) of inputs (black circles; non-Gaussian), and output distributions (synaptic conductances) for a non-divisive (perfect) differentiator (red crosses) and a divisive (perfect) differentiator (blue crosses). For comparison, a Gaussian distribution is shown (solid black line). C: Estimated kurtosis from the distributions in B for different scales of the natural input distribution. Note that the output of the PI circuit achieves a synaptic conductance distribution that is much closer to a Gaussian. A Gaussian has a kurtosis of 3 (dashed line).

It was suggested previously that divisive normalization could gaussianize the non-Gaussian statistics of natural signals, however, without discussing a concrete neural mechanism [38]. We asked whether the PI circuit motif could transform the statistics of natural signals to a Gaussian-like form. To test this, we generated a temporally correlated noise signal with statistics following a Student-t distribution to approximate the statistics of natural signals, and used it as input to the PI circuit. When comparing the distribution of the resulting synaptic conductances (that is, $g^{\mathrm{EE}}$) with that of input signals, we found that, if PI is present, the output becomes indeed more similar to a Gaussian distribution (as seen by the reduced kurtosis of the output in respect to the input; see Fig. 6B), while a circuit without divisive inhibition is not able to gaussianize the natural input statistics (see Fig. 6A). In summary, the PI circuit not only can handle input signals over a wide dynamic range, it also squashes natural input signals to become more Gaussian; a potential important pre-processing operation for subsequent neural information processing.

### Adaptive perfect differentiation

**Fig 7:**
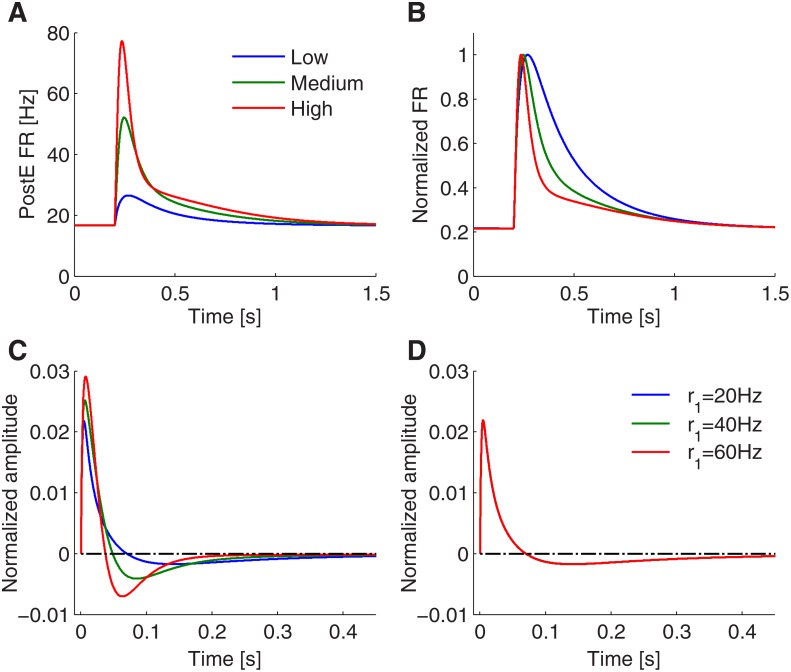
Contrast adaptive perfect differentiation. A: **PostE** neurons’ transient firing rate response for different input contrasts (low, medium, and high). B: Normalizing the **PostE** neurons’ response (of panel A) to the peak amplitude reveals the contrast dependent dynamics of the decay. C: Estimated filters for the three stimulus contrasts. Note that the width of the integration window (positive part) is stimulus contrast dependent. D: When varying the baseline activity of **PreE** neurons (*r*_1_) while fixing the contrast to 2 (by setting *r*_2_ accordingly), the estimated filter remains identical. Thus, the estimated filter of the PI circuit is intensity invariant. Parameters: *r*_1_ = 20 Hz, *r*_2_ = 60 Hz, 140 Hz and 220 Hz.

We have shown that the PI-circuit can achieve perfect differentiation. Next, we analyzed the dynamics of the circuit’s transient response in more detail. In particular, we examined the adaptivity of the **PostE** neurons’ average response to the stimulus contrast, i.e. (*r*_2_ − *r*_1_)/*r*_1_. In Fig. 7A, we plot the transient response of the circuit to low, medium, and high contrasts, and then normalize the peak amplitudes of the transient dynamics (Fig. 7B), to better compare the time courses of the responses. The **PostE** firing rate decays to its stationary state within a shorter time period if the contrast is high, whereas the decay becomes markedly slower for low contrast stimuli. Similarly, if we estimated the temporal filter of the neural circuit (see Methods), we found that the temporal filter has a longer integration window for low contrasts, and a shorter integration window for high contrasts (see Fig. 7C). While varying the baseline activity without changing the contrast, the shape of the temporal filter does not change, thus showing a baseline activity invariant response (see Fig. 7D). This form of stimulus dependent transient response dynamics is consistent with observations in retina ganglion cells, where responses similarly adapt to different contrasts [15, 33, 42].

The adaptive behavior can be explained mathematically by examining the derived mean-field dynamics (see Methods). We found that the **PostE** firing rate is mainly determined by the slow-varying dynamics of the vesicle resource *x*, the vesicle release probability *u*, and effective spike amplitude *p*. The time constant for *x* and *u* can be respectively written as

$$\begin{array}{c} \tau_{x}=\frac{\tau_{D}}{1+\tau_{D}upr} \end{array}$$ (34)

$$\begin{array}{c} \tau_{u}=\frac{\tau_{F}}{1+U\tau_{F}pr}. \end{array}$$ (35)

**Fig 8:**
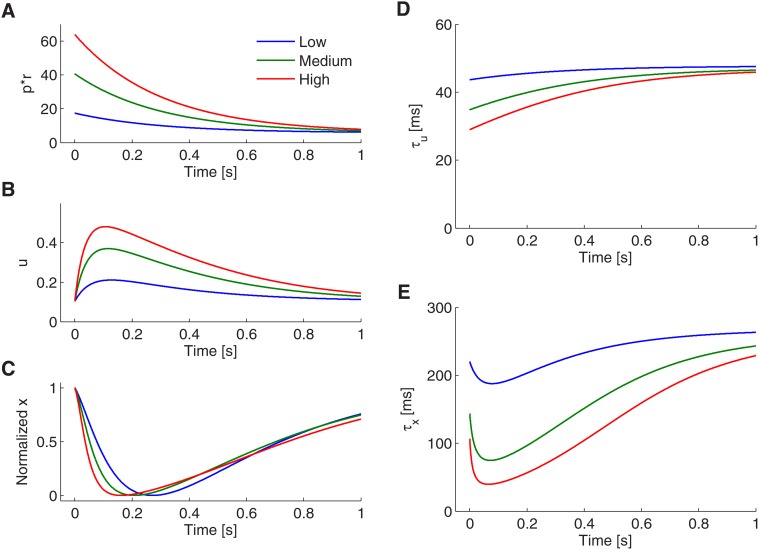
Contrast dependent PostE responses can be explained by dynamics of vesicle resource and release probability. A–C: Transient responses of the effective input rate *pr* (A), release probability *u* (B) and the normalized vesicle resource *x* (C) to different input contrasts. D–E: Change of the time constants of the release probability *τ*_*u*_ (D) and vesicle resource *τ*_*x*_ (E) in response to different inputs contrasts. Note that as the time constant of release probability depends on effective input rate *pr* (dynamics shown in A), dynamics of release probability is therefore contrast dependent (see B). Also the time constant of vesicle resource depends on dynamics of effective input rate and release probability, so that the vesicle resource exhibits contrast dependent responses (see C).

Note that these effective time “constants” for the vesicle release probability *τ*_*u*_ and vesicle resource *τ*_*x*_ depend on the sensory input *r* and thus change dynamically when the input changes abruptly. When the input rate *r* is fixed, the system is at a steady state and the time constants are static as well. However, for an abrupt input change, *r*_1_ → *r*_2_, the time constants for the release probability *τ*_*u*_ will increase slowly according to the change in the PI variable *p*, which in case of perfect differentiation decreases smoothly from *p* ∝ 1/*r*_1_ to *p* ∝ 1/*r*_2_ on a time scale of the order of *τ*_*p*_ (see Fig. 8A). Consequently, the larger the increase of the input rate is (i.e. the larger *r*_2_), the smaller is *τ*_*u*_ at the initial stage after the stimulus change (see Fig. 8D). However, the contrast dependence of the release probability time constant is small compared to that of the vesicle resource *τ*_*x*_. This is because the **PreE**-to-**PostE** synapse is depression dominant in case of differentiation; it is thus generally *τ*_*u*_ < *τ*_*x*_. The strong contrast dependence of *x* can be seen by observing in Eq. 34 that *τ*_*x*_ not only depends on the product *pr* but additionally on the release probability *u* itself. Since the release probability varies greatly with contrast level (see Fig. 8B), so does the time constant of *x* (see Fig. 8E). Note that the time constant *τ*_*x*_ can initially vary more than five-fold for different contrast levels (see Fig. 8E at time 50ms after stimulus change). In summary, the adaptive transient dynamics for different stimuli contrasts are mostly caused by the differential dynamics of the vesicle resource variable *x*. Thus the short-term plasticity of the **PreE**-to-**PostE** synapse causes the system to be adaptive to different contrast levels.

**Fig 9:**
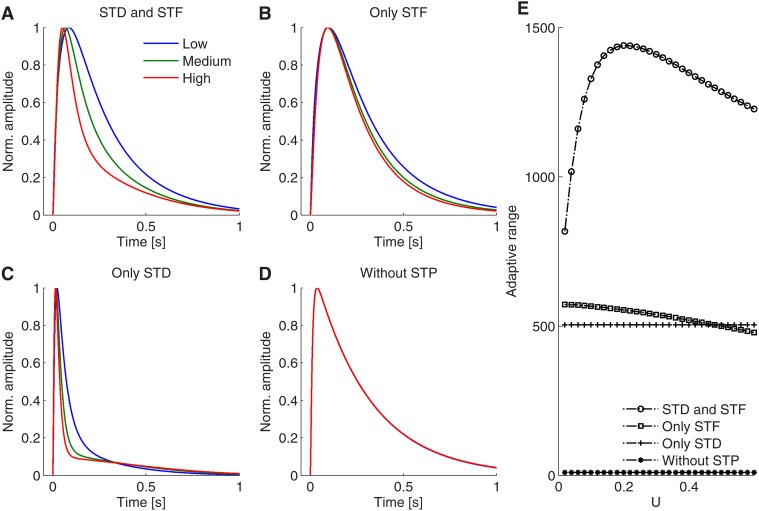
Adaptive ranges for different STP settings of the PreE-to-PostE synapse in the PI circuit. A–D: Adaptive range when STD or STF (or both) are blocked off (as indicated in the title). In simulations, when blocking STD or STF (or both), we set vesicle resource (release probability) to be a constant value when input rate is fixed. The adaptive range is defined by the difference of the normalized response areas between low and high contrasts. E: Changes in the adaptive range when varying the short-term depression strength *U* in the corresponding cases from A–D.

To further investigate how this adaptive response is affected by either synaptic short-term depression (STD) or short-term facilitation (STF), we compared the adaptive range of neural responses when one of STD or STF (or both) are blocked (see Fig. 9). Using the difference of the area under the normalized response between a high and a low contrast as a measure for the adaptive range, we found that both, STD and STF contribute to the adaptive behavior. In Fig. 9A–D normalized neuronal responses in all four STP conditions are shown. If both, STD and STF, are present, the adaptive range is largest, if only STD or STF is present, adaptivity is reduced. If neither STD nor STF is present, adaptation vanishes. The enlarged adaptive range in case of both STD and STF originates from the cooperative transient dynamics in response to the stimulus as detailed above. By tuning the parameter *U*, the size of the adaptive range reaches a maximal value for moderate strength of synaptic facilitation (see Fig. 9E).

### Contrast dependent differentiation: comparison to experiments

**Fig 10:**
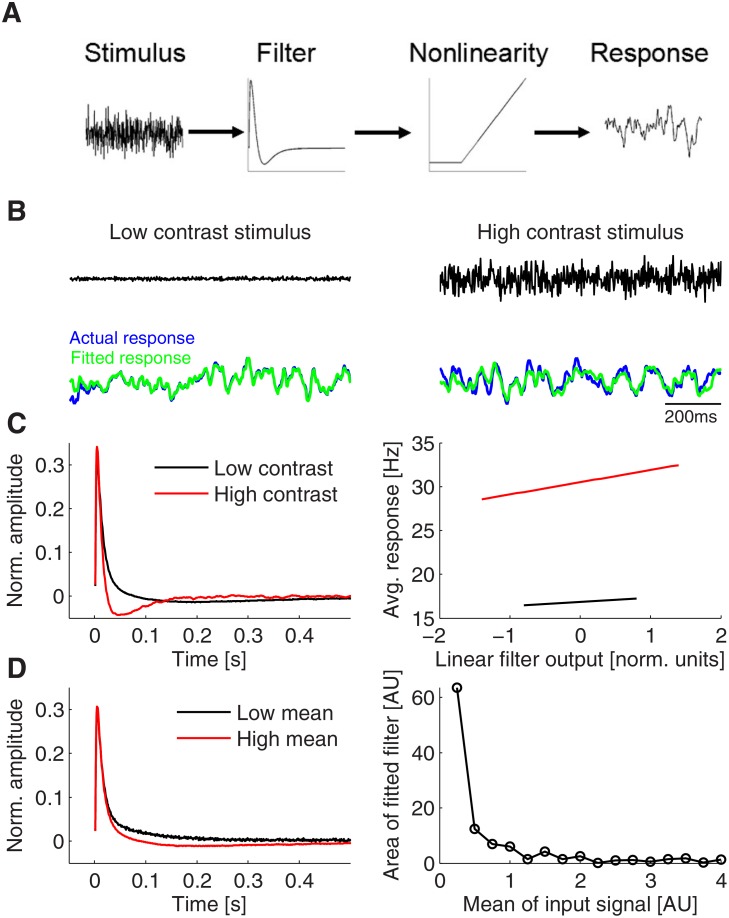
Linear-nonlinear characterization of neuronal responses. A: Scheme of the linear-nonlinear model to fit **PostE** responses. The stimulus is first convolved with a linear filter, then the filter response is passed through a fixed nonlinearity to fit the actual responses of the dynamical system. B: Actual **PostE** responses plotted together with the fit of the linear-nonlinear model at low and high contrast input. C: Estimated filters for low and high contrast (left) and the corresponding non-linearity (right) of the fitted linear-nonlinear model. To show the non-linearity, the average response of the linear-nonlinear model are plotted versus the output after the linear stage only. Note that the filter has a narrower integration window and that the non-linearity shifts to higher values for high contrast stimuli. D: Estimated filters for inputs with low and high mean (with identical contrasts). Note that the activation threshold of the inhibitory population causes the system to integrate for weak inputs, as indicated by the large positive area of the estimated filter for weak stimuli (right). The filter area approaches to zero for inputs with high mean, as expected for a perfect differentiator.

We have found that the adaptive PI-circuit response is contrast dependent, with slower transient activity decay for low contrast inputs than for high contrast inputs. This adaptivity arises from the input dependent transient activity of the vesicle resource and the release probability. Experiments have measured the linear filtering and nonlinearity property of different cell types in the retina [15]. It was found that ganglion cells exhibit strong adaptivity of their filtering properties, while other types of cells in the retina show relatively smaller or no adaptivity, e.g., horizontal, bipolar and amacrine cells. Currently, there is a debate whether this contrast adaptability originates from an intra-cellular or from an inter-cellular synaptic mechanism [43–46]. Our PI-circuit is well applicable to this retinal circuit, since it is known that the feedforward connections of bipolar cells onto ganglion cells are presynaptically inhibited by amacrine cells [17, 18]. To test whether the experimental observations could be reproduced in our model, we artificially applied two stimuli with equal mean intensity but different variance (i.e. the definition of two types of contrast levels in the experiments) to our adaptive PI-circuit. Using a linear- nonlinear model to fit the circuit’s input-output relationship (Fig. 10A–B; see Methods for details), we found that the resulting linear temporal filter has a narrower integration window for high contrast inputs and a wider integration window for low contrast inputs (Fig. 10C), reproducing the experimental observations [15, 46]. Note that this adaptivity is beneficial for optimal information transmission in face of noise [47]. Moreover, the nonlinearity estimated from the model was upwards shifted during the transition from low to high contrast inputs (Fig. 10C), consistent with the measurements of the nonlinearity in the ganglion cells membrane potentials [15].

In summary, the STP-PI circuit seems to well reproduce the experimental observations suggesting that the observed adaptability in the retinal circuit might well arise from short-term plasticity of synaptic transmission and not necessarily from other intra-cellular mechanisms.

## Discussion

We compared a number of simple circuit motifs in their capabilities to respond adaptively to sudden changes in the input. We found that unlike other feedforward inhibition motifs that have been described previously [6, 7], our newly proposed PI-circuit featuring both pre-synaptic inhibition and short-term plasticity acts like a contrast adaptive perfect differentiator. To our knowledge, a biological plausible circuit that achieves contrast adaptive perfect differentiation of input signals has not been described before.

Presynaptic inhibition is a form of synaptic inhibition that is mediated via an axo-axonic synapse complex [24]. Inhibition of the presynaptic terminals of the excitatory synapse is caused by the activation of ligand-gated chloride channels triggered by the release of neural transmitter GABA that is secreted from an inhibitory synaptic terminal in close vicinity of an excitatory synapse. In our model, we assume that the increase of chloride conductance in the excitatory terminal reduces the effective amplitude of the action potential and thus potentially shunts the current flowing into the presynaptic terminal [24, 48].

In our analysis, we found that whether feedforward inhibition is mediated pre- or post-synaptically is a crucial difference. Although both mechanisms can show perfect differentiation, the way of establishing the balance of excitation and inhibition is qualitatively different. In the presynaptic feedforward inhibition circuit, excitatory current is *divisively* balanced by inhibitory inputs, while for postsynaptic feedforward inhibitory circuit, excitation and inhibition are *subtractively* balanced. Thus, in contrast to the common notion of balanced excitation and inhibition in neural circuits [49, 50], in the PI circuit the ratio of excitatory and inhibitory synaptic currents is balanced, and not their sum.

Divisive or subtractive balance differs in several aspects in regard to their signal processing properties. First, presynaptic inhibition maintains the vesicle resource and release probability at a proper value regardless of the input intensity, thus enabling efficient utilization of vesicle resource without potential depletion of resources for high input intensities. In contrast, in case of postsynaptic inhibition, vesicle resources will decrease in proportion to the presynaptic firing rate and thus cannot be maintained. Second, when the excitatory synapse exhibits short-term depression, the feedforward inhibitory pathway would also need an identical form of short-term depression, i.e. the very same depression time constant, to guarantee a detailed balance of excitation and inhibition. This seems highly unlikely to be realizable in practice. Finally, because of the divisive nature of the presynaptic inhibition, the PI circuit can reduce higher-order statistics of sensory inputs (as shown in the Results, Fig. 6), while a subtractive balanced circuit cannot. Taken together, the PI circuit seems to be a better candidate for implementing differentiation in early sensory systems.

### Perfect differentiator

In the PI circuit, perfect differentiation is largely due to presynaptic modulation of the excitatory synaptic transmission efficacy. To achieve perfect differentiation in the circuit, the strength of the presynaptic inhibition has to be reversely proportional to the inhibitory activity. In this case, excitation and inhibition is perfectly balanced with each other. It has been reported that the balance of excitation and inhibition could be learned in a feed-forward network with inhibitory synaptic plasticity [51, 52]. Thus it is conceivable that the strength of PI could be learned as well to ensure a divisive balance in excitation and inhibition. The divisive nature of presynaptic inhibition ensures that the circuit response scales with the baseline activity, thus obeying the Weber-Fechner law. In our model, presynaptic inhibition modulated the effective amplitude of the generated spikes. This mechanism is different from previous models [9], where it was postulated that Weber’s law results from the residual vesicle resource in the presynaptic site.

Responses according to the law of Weber-Fechner ensure that the dynamic range of input signals is compressed into the dynamic range of single neurons. The discussed presynaptic inhibition mechanism ensures a dynamic range compression by relying on a specific connectivity between different neuron types, namely a specific type of feed-forward inhibition. It was reported that the dynamic range of input signals might also be compressed through single neuron properties, for instance by active dendrites or electrical synapses [53, 54]. Since signal compression is essential for coding a high dynamic range, sensory system might rely on multiple mechanisms. The relative importance of one or the other mechanism will likely depend on the specific sensory system in question and can only be estimated based on experimental observations.

Experimentally, it was found that sensory neurons act as differentiators in response to strong enough sensory inputs but can also act as integrators for weak inputs [5, 15]. This dual-use of sensory neurons could be important for neural information processing. For weak inputs, the signal-to-noise ratio (SNR) is low. Sensory neurons thus need to integrate over a longer time period to collect enough information. For strong inputs with high SNR on the other hand, the sensory neurons should act as a differentiator to specifically detect changes in the sensory inputs [5]. This transition from an integrator to a differentiator depending on the stimulus strength can be achieved by the proposed PI circuit when considering a threshold for the activation of the inhibitory neurons. If the spiking threshold of the inhibitory neurons were relatively large, the feedforward inhibition pathway would not be activated for weak inputs, so that the neural response was proportionally to the signal intensity and could thus integrate weak sensory inputs. However, a moderately strong sensory input would activate the inhibition pathway, so that the circuit would act like a prefect differentiator instead, detecting only changes in the sensory inputs (see Fig. 10D).

### Contrast adaptivity

Our results suggest a novel mechanism for contrast adaptation in early sensory system. In particular, we found that STP mediated the contrast adaptivity of the PI-circuit. STP has been only recently more thoroughly investigated theoretically and STP was suggested to play various important computational roles, e.g. in the generation of memory traces [55], persistent activity [28, 56], and for generating slow oscillatory activity [28]. Interestingly, we found that the adaptive range of the filter can reach a maximum for an intermediate value of the strength of facilitation. We found by fitting a linear-nonlinear model that the PI circuit adapts its filter similarly to experimentally observed membrane potentials in ganglion cells in the retina. In our simple neuron model, spiking activity is directly proportional to the membrane potential, so that the spiking activity shows the same behavior as the membrane potentials. However, in experiments ganglion cells spiking activity are not directly proportional to the membrane potentials. This difference is due to an over-simplification of our **PostE** neuron spiking mechanism, rather than to an inapplicability of the PI circuit to the experimental data. When our circuit model would be extended to additionally include postsynaptic shunting inhibition [32] and neural dynamics would additionally include a slow intracellular hyperpolarization current, we expect that the experimentally observed gain change and rightward shift of the estimated nonlinearity of spiking activity in ganglion cells would likely be also reproduced.

In summary, our results indicate that the experimental findings of contrast adaptivity might indeed be a result of the plasticity of synaptic contacts rather than caused by other intra-cellular mechanism. Whether this suggestion is correct could be tested in future experiments.
